# EGFR gene methylation is not involved in Royalactin controlled phenotypic polymorphism in honey bees

**DOI:** 10.1038/srep14070

**Published:** 2015-09-11

**Authors:** R. Kucharski, S. Foret, R. Maleszka

**Affiliations:** 1 Research School of Biology, The Australian National University, Canberra ACT 0200, Australia

## Abstract

The 2011 highly publicised *Nature* paper by Kamakura on honeybee phenotypic dimorphism, (also using *Drosophila* as an experimental surrogate), claims that a single protein in royal jelly, Royalactin, essentially acts as a master “on-off” switch in development via the epidermal growth factor receptor (AmEGFR), to seal the fate of queen or worker. One mechanism proposed in that study as important for the action of Royalactin is differential *amegfr* methylation in alternate organismal outcomes. According to the author differential methylation of *amegfr* was experimentally confirmed and shown in a supportive figure. Here we have conducted an extensive analysis of the honeybee *egfr* locus and show that this gene is never methylated. We discuss several lines of evidence casting serious doubts on the *amegfr* methylation result in the 2011 paper and consider possible origins of the author’s statement. In a broader context, we discuss the implication of our findings for contrasting context-dependent regulation of EGFR in three insect species, *Apis mellifera*, *D. melanogaster* and the carpenter ant, *Camponotus floridanus*, and argue that more adequate methylation data scrutiny measures are needed to avoid unwarranted conclusions.

In the social honey bee *Apis mellifera,* two alternative female phenotypes, long-lived fertile queens and short-lived sterile workers are produced via differential feeding with a diet known as royal jelly (RJ)^1^,^2^,^3^. This complex, still poorly understood nutrition contains various ingredients^4^,^5^ including carbohydrates, vitamins, unusual lipids, antimicrobial agents, epigenomic modifiers such as histone deacetylases inhibitors (HDACs)^6^, as well as many other less characterised compounds^4^,^5^. The bulk of RJ is formed by several Major Royal Jelly Proteins (MRJPs) that appear to be unique to Hymenoptera^2^. MRJPs evolved from the insect Yellow protein family that has its origins in bacteria^2^,^7^. No relatives of MRJPs or Yellow proteins have been found in modern vertebrates, but a Yellow-like protein is encoded by the genome of a chordate *Branchiostoma floridae* (GenBank XP_002607604). The remarkable developmental potency of RJ has been attributed to a synergistic effect of many if not all of its components acting as activators of signalling pathways via threshold based changes in metabolic flux and epigenomic modifications^8^,^9^,^10^,^11^. This view has been somewhat obscured by the 2011 study claiming that one of the MRJPs, labelled Royalactin, is capable on its own to drive all the changes needed to make a queen bee^12^ effectively reducing the entire process of queen development to the vagaries of one protein. Furthermore, treating *Drosophila* with Royalactin appears to increase body size and ovary development in female flies with the Canton S genetic background^12^. In order to reconcile those findings with prior evidence implicating DNA methylation in queen development^13^,^14^, Kamakura conducted DNA methylation analysis in the genomic region encoding AmEGFR. In one of the Figures (S34)^12^ he shows that “the overall level of methylation of *amegfr* in larvae reared with RJ (5%), which develop into queens, was decreased as compared with that in larvae reared with 40-30d RJ (57%), which emerge as workers. Similar results were observed in queen larvae and worker larvae reared in hive”^12^. Since this result stands in stark contrast to the absence of *amegfr* methylation in published honey bee methylomes^9^,^15^,^16^, including larvae of the same age as in the Kamakura paper^12^, we have conducted detailed analyses to re-examine the methylation status of *egfr* in *Apis mellifera*. We show not only that *amergf* is never methylated but has a high GC content that is consistent with non-methylated genes, and consider the origins of this unfeasible result in the Kamakura study.

## Results

**Figure 1:**
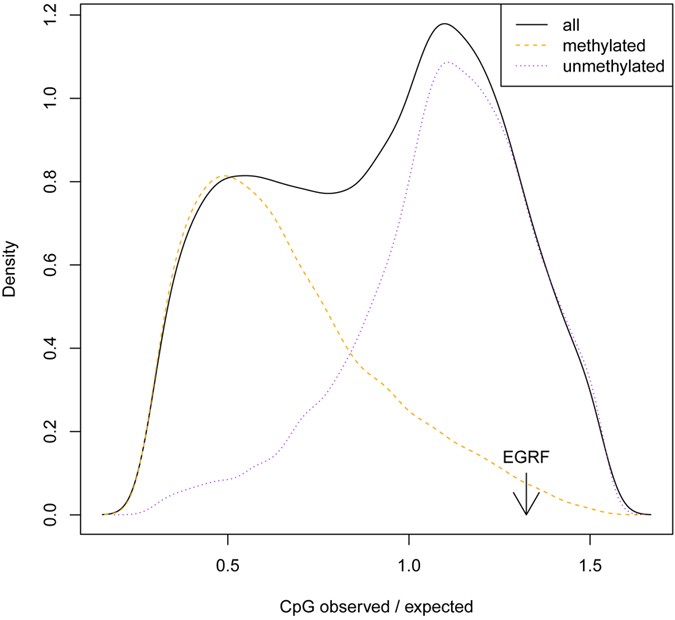
CpG (observed/expected) bias of protein-coding regions in the honey bee genome. The o/e value of 1.104 for the analysed *egfr* region is indicated with the arrow. The o/e value for the whole gene is even higher (1.324).

Our desire to meticulously examine the levels of *egfr* methylation in *Apis* was inspired by the absence of any methylated CpGs in this locus in the published methylomes from both queens and workers Table 1). In particular, the larval methylomes^9^ representing queen and workers of the same 96 hrs stage of development as in the Kamakura’s paper^12^ were indicative of a potential problem. In addition, no methylation of *amegfr* has been found in several brain methylomes generated by two independent labs^15^,^16^. For comparison, we have contrasted sequencing coverage and methylation levels for *egfr* with a consistently methylated gene *nadrin* using 11 methylomes representing different tissues, cell types and developmental stages (Table 1). We also have noted that the 736 bp region of *amegfr* deemed to be methylated in the Kamakura study^12^ is rich in CpG dinucleotides, which is untypical for methylated genes in *Apis* that are CpG-depleted because of the known tendency of 5-methyl cytosines to be converted into thymines^8^,^15^,^17^,^18^. The ratio of observed to expected (o/e) CpG dinucleotides is 1.104 in the 736 bp fragment and 1.324 for the whole gene. Such values are associated with non-methylated genes in *Apis*^8^,^15^ (Fig. 1) that can be easily separated from methylated genes in a bimodal distribution of the o/e frequency of CpGs in all annotated genes. However, given the low sequencing depth of genome-wide methylomes and the possibility that *amegfr* may be methylated in a restricted number of cell types that could have been picked by chance in the 2011 study, we have decided to reproduce the original low-depth plasmid sequencing experiment using ultra-deep sequencing of the same genomic region^12^. Unfortunately, we have failed to amplify the 736 bp DNA fragment following the conditions described in the 2011 paper. This is not entirely surprising as it is nearly impossible to amplify DNA fragments of such length from bisulfite treated DNAs. The DNA fragmentation during the treatment^19^ leads to a practical upper size limit of the PCR amplicon ~400–500 bp, and the need for nested primers and a second round of PCR is often essential^20^. Instead, we used two sets of outer, and two sets of nested primers to amplify two overlapping amplicons (A1 = 440 bp and A2 = 441 bp) covering the supposedly methylated region of *amegfr*. To properly design our amplicons we had to re-annotate the old erroneous EGFR gene model used in the 2011 study (see Figure S1 for details). These amplicons have been processed and barcoded for ultra-deep sequencing with the NEB kit for Illumina libraries. Both libraries A1 and A2 have been sequenced together with additional libraries representing confirmed methylated genes on Illumina MiSeq using the Reagent Kit v3 that generates up to 25 million reads (15Gb) of 2 × 300 bp paired end reads.

**Figure 2:**
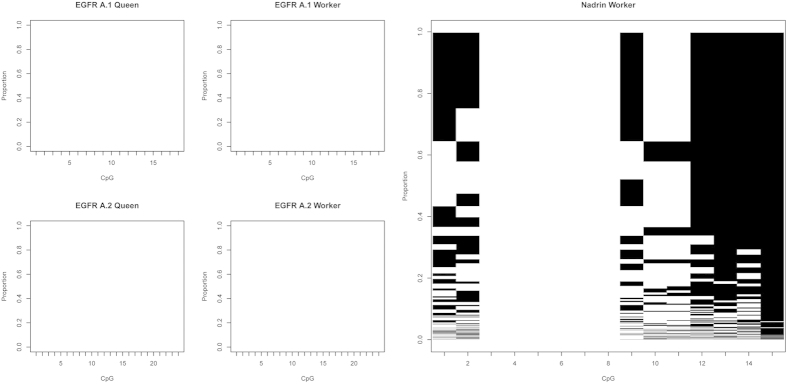
Methylation patterns in *egfr* and *nadrin* revealed by deep amplicon sequencing. Each row represents a methylation pattern (black: methylated CpGs, white: not methylated CpGs), the height of each pattern is proportional to the pattern’s abundance. Two EGFR amplicons (A1 and A2) were amplified from 96 hrs old queen and worker larvae. One *nadrin* amplicon was amplified from 96 hrs worker larvae that have been shown in the Kamakura paper to have elevated methylation levels. After normalising pattern frequencies using MPFE^21^, no methylation was detected in *egfr*, whereas several distinct and highly abundant methylation patterns are detected in the *nadrin* amplicon. The pattern proportions are sorted from the most abundant at the top to the least abundant at the bottom.

As shown in Table 2 even with a depth of over 330,000 reads per amplicon virtually no methylation of EGFR in larval samples has been detected. The methylation pattern counts were normalised with MPFE^21^ in order to eliminate most of the spurious patterns caused by sequencing errors and incomplete bisulfite conversion. As a result, for both *amegfr* amplicons A1 and A2, over 99.99% of reads were classified as not methylated. The very few methylated patterns observed in *egfr* are most likely technical noise and represent less than 0.01% of the total counts. Failure to detect methylation in the *egfr* region is unlikely to be a PCR artefact because methylated DNA tends to be over-represented in bisulfite sequencing data^22^. With this depth of sequencing it is likely the methylation patterns in essentially every cell type can be visualised as illustrated by a genuinely methylated gene, *nadrin* (Fig. 2). In larval samples, 157 distinct methylation patterns were observed in this amplicon after removing the spurious patterns. Whether or not these patterns represent unique epigenetic signatures of 157 cell types in growing larvae remains to be established.

## Discussion

Our findings showing that the honey bee *egfr* gene belongs to the non-methylated category make the result shown in the Kamakura paper^12^ impossible to reproduce. There are three lines of evidence questioning the correctness of the original claim. First, on the basis of its high CG content, *egfr* is bioinformatically predicted to be non-methylated. Second, genome-wide methylation profiles in larvae, brains and embryos from both queens and workers show no evidence of methylation. Third, our new data using ultra-deep amplicon sequencing failed to detect any sign of methylated cytosines in larval samples. At this stage the source of this questionable result is unclear. One possibility is that the original DNA samples were not properly converted with bisulfite thus leading to a false impression that some cytosines in *egfr* were protected by methyl tag. This problem could have been exaggerated by omitting nested primers that typically are used to improve the recovery of AT-rich methylated amplicons from BS-treated DNA. However, the increase of *amegfr* methylation only in worker larvae in two situations shown in the Kamakura paper (Figure S34) suggests that such an experimental clarification is unlikely. Whatever the reason for this doubtful result is, our findings have important consequences for understanding how conditional phenotypes are implemented in various lineages by engaging cell surface signalling via EGFR and its growth factor ligands.

While it is not surprising that this important cell surface receptor has been implicated in the queen crafting process in honey bees and growth regulation in *Drosophila*, it is evident that its regulation is not contingent on DNA methylation. Although a queen bee can be experimentally induced by silencing *de novo* DNA methylation in newly hatched larvae by means of RNAi approach^13^, such treatment is pleiotropic and affects hundreds of genes and pathways relevant to nutritional sensing, such as for example, the TOR/insulin network, and importantly, Anaplastic Lymphoma Kinase that has the capacity to directly induce downstream events by bypassing insulin signalling^9^,^23^. A queen phenotype can also be induced by interfering with the expression of genes belonging to the TOR/insulin network^24^ and possibly other manipulations affecting nutritional signalling. For example, royal jelly is exceedingly rich in both methionine and sources of methyl groups (choline) and some of its MRJPs are unusually rich in this essential amino acid, providing substrates for methylation activities. In this context, the finding by Grandison *et al.*^25^ that in *Drosophila*, methionine alone was necessary and sufficient to increase fecundity as much as did full feeding, but without reducing lifespan, is striking. Although the effects of RJ on honey bee larval growth are still not fully appreciated, there is little doubt that methylation changes and diet are clearly linked. It may be prudent to more closely examine the relationships between caloric restriction, methylation and foods rich in methionine, acetylcholine with longevity and fecundity in both *Apis* and *Drosophila*. Royalactin is simply one of very many components that contribute to network flux. Obviously, it has a defined role in this process, but until all components of RJ are better understood its exclusive role *in vivo* should be considered with caution.

It is most interesting that *egfr* methylation has recently been implicated in generating quantitative variation in size of the carpenter ant^26^ allowing for comparative analyses of a highly conserved EGFR in three insect species in the epigenetic context. *Drosophila* lost its DNA methyltransferases and has to use other epigenetic mechanisms for EGFR regulation, whereas in two Hymenopterans with the complete DNA methylation toolkit, ants and honey bees, only one recruits methylation to regulate *egfr*. One possible explanation for such a contrast between ants and honey bees is that methylation in the carpenter ant is utilised as an indirect modulator of *egfr* for continuous size variations, whereas in *Apis* such a flexible epigenomic modification of *egfr* would not be desirable for the proper development of the focal individual that is critical for the colony. These comparative analyses underscore the inherent pitfalls in data transferability between different species at particular levels. In spite of a high level of EGFR conservation, both structural and functional, its context-dependent regulation appears to be driven by distinct mechanisms in insect species. An organism can utilise many different cellular systems to accomplish the same end result as long as it is the desired phenotypic outcome. Indeed, to solve the same problem many different designs can be constructed even from heteromorphic, but functionally similar elements because of the high level of degeneracy in biology^27^.

In conclusion, we argue that methylation data claimed to be relevant to specific biological processes need to be supported by in-depth analyses using newer, high resolution methods. However, whatever platform one utilizes for methylomic insights, all of them have to be properly scrutinised to answer the critical question: how relevant is the observed differential methylation to the phenomenon under investigation?

## Materials and Methods

### DNA bisulfite conversion and amplicon preparation

1.5 μg of larval genomic DNA^28^ was bisulfite converted using the QIAGEN Epitect® Bisulfite Kit, as per the manufacturer’s protocol^29^. We routinely perform two consecutive treatments to avoid incomplete conversion especially in GC-rich regions. The converted DNA was amplified via a nested PCR reaction with *amegfr* specific primers (see Table S2 for BS-seq primers). The PCR products were purified utilising Agencourt® AMPure® XP PCR Purification system (Beckman Coulter).

### NGS library preparation

Libraries were prepared from 500–600 ng of each amplicon utilising the NEBNext® DNA Library Prep Master Mix for Illumina®, and NEBNext® Multiplex Oligos for Illumina® Index Primers Set 1 and Set2 (New England Biolabs). Size selection of adaptor ligated DNA was performed using Agencourt AMPure XP beads (Beckman Coulter), with the bead:DNA ratio of the first bead selection 0.9X, followed by a second bead selection with bead:DNA ratio at 0.2X (not sure about the ratios, I do not think this is necessary). Each library was eluted in 30 μL of 0.1X TE, library size confirmed via agarose gel electrophoresis (we used Caliper LabChip GXII and HT DNA High Sensitivity Assay), and diluted to a final concentration of 4 nM.

### NGS MiSeq sequencing

Next generation sequencing was performed on Illumina MiSeq instrument using MiSeq Reagent Kit v3 (Illumina) and 600 cycles. PhiX spike was added at 5% concentration as recommended by Illumina for low-diversity libraries.

### Analysis of bs-seq results

For each individual analysed the frequency at which a mCpG occurred was calculated across all reads using custom Python scripts and open-source software. The process comprised of two steps. In the first, pairs of reads with the 30 nucleotide sequence starting at position 4 matching exactly the last 30 nucleotides of the primers used for nested amplicon PCR were extracted from FASTQ files, aligned with *in silico* bisulfite-converted genomic template using MUSCLE^29^, overlapping regions (if any) were proportionally truncated and, after removing all aligner-introduced gaps, both reads were combined into one continuous sequence and appended to a to a separate file (“extract file”) for each amplicon and each library/sample. In addition, a quality filter was applied, rejecting all sequences shorter than 90% of the length of the template or containing in excess of 5% gaps. In the second step, batches of sequences from the “extract” files were re-aligned with the template using MUSCLE (to eliminate any potential positional errors introduced by read indels), the aligned template sequence was used to calculate positional information of all the expected CpGs and SNPs, and the positional data were used to score methylation status [ie. 0 for T and 1 for C occurring at a CpG position] for each combined read pair. The data were next appended to a separate table for each amplicon and each library/sample. The final tables were used to calculate and graph amplicon methylation data using MPFE^21^.

## Additional Information

**How to cite this article**: Kucharski, R. *et al.* EGFR gene methylation is not involved in Royalactin controlled phenotypic polymorphism in honey bees. *Sci. Rep.* **5**, 14070; doi: 10.1038/srep14070 (2015).

## Supplementary Material

Supplementary Information

## Figures and Tables

**Table 1:** Sequencing coverage and *egfr/nadrin* methylation in the honey bee whole-genome bisulfite sequencing projects.

**Dataset**	**Result**	**EGFR**	**NADRIN**
Queen and worker larval heads^9^	mean coverage*	34.5	207.6
	methylation	0%	43.6%
Queen and worker adult brains^15^	mean coverage	22.3	16.2
	methylation	0%	40.9%
Haploid and diploid embryos (various stages of development)**	mean coverage	16.1	23.3
	methylation	0%	28.7%
Forager brains^16^	mean coverage	7.0	6.7
	methylation	0%	42.9%
Queen brains^16^	mean coverage	12.7	11.5
	methylation	0%	46.9%
Reverted nurses brains^16^	mean coverage	5.5	4.3
	methylation	0%	40.5%
Worker brains^16^	mean coverage	11.2	11.1
	methylation	0%	43.0%

*average from queen and worker libraries, **in preparation.

**Table 2:** Sequencing coverage and *egfr* methylation level in MiSeq high-throughput amplicon experiment.

**Dataset**		**Amplicon 1**	**Amplicon 2**
96 hrs Queen larvae	Reads per amplicon	1,873	339,596
	methylation	0%	<0.01%
96 hrs Worker larvae	Reads per amplicon	4,168	334,837
	methylation	0%	<0.01%

## References

[b1] Maleszka R. Epigenetic integration of environmental and genomic signals in honey bees: the critical interplay of nutritional, brain and reproductive networks. Epigenetics 3, 188–192 (2008). 18719401 10.4161/epi.3.4.6697

[b2] Drapeau M. D., Albert S., Kucharski R., Prusko C. & Maleszka R. Evolution of the Yellow/Major Royal Jelly Protein family and the emergence of social behavior in honey bees. Genome Res 16, 1385–1394 (2006). 17065613 10.1101/gr.5012006 PMC1626640

[b3] Weaver N. Rearing of Honeybee Larvae on Royal Jelly in the Laboratory. Science 121, 509–510 (1955). 17817382 10.1126/science.121.3145.509

[b4] Shuel R. W. & Dixon S. E. Studies in the mode of action of royal jelly in honeybee development II.Respiration of newly emerged larvae on various substrates. Can J Zoolog 37, 803–813 (1959).

[b5] Rembold H. & Dietz A. Biologically active substances in royal jelly. Vitamins & Hormones 23, 359–382 (1965). 5326344 10.1016/s0083-6729(08)60385-4

[b6] Spannhoff A. *et al.* Histone deacetylase inhibitor activity in royal jelly might facilitate caste switching in bees. Embo Reports 12, 238–243 (2011). 21331099 10.1038/embor.2011.9 PMC3059907

[b7] Maleszka R. & Kucharski R. Analysis of Drosophila yellow-B cDNA reveals a new family of proteins related to the royal jelly proteins in the honeybee and to an orphan protein in an unusual bacterium Deinococcus radiodurans. Biochem Biophys Res Commun 270, 773–776 (2000). 10772900 10.1006/bbrc.2000.2506

[b8] Foret S., Kucharski R., Pittelkow Y., Lockett G. A. & Maleszka R. Epigenetic regulation of the honey bee transcriptome: unravelling the nature of methylated genes. BMC Genomics 10, 472 (2009). 19828049 10.1186/1471-2164-10-472 PMC2768749

[b9] Foret S. *et al.* DNA methylation dynamics, metabolic fluxes, gene splicing, and alternative phenotypes in honey bees. P Natl Acad Sci USA 109, 4968–4973 (2012). 10.1073/pnas.1202392109 PMC3324026 22416128

[b10] Metallo C. M. & Vander Heiden M. G. Metabolism strikes back: metabolic flux regulates cell signaling. Gene Dev 24, 2717–2722 (2010). 21159812 10.1101/gad.2010510 PMC3003187

[b11] Wellen K. E. *et al.* ATP-Citrate Lyase Links Cellular Metabolism to Histone Acetylation. Science 324, 1076–1080 (2009). 19461003 10.1126/science.1164097 PMC2746744

[b12] Kamakura M. Royalactin induces queen differentiation in honeybees. Nature 473, 478–483 (2011). 21516106 10.1038/nature10093

[b13] Kucharski R., Maleszka J., Foret S. & Maleszka R. Nutritional control of reproductive status in honeybees via DNA methylation. Science 319, 1827–1830 (2008). 18339900 10.1126/science.1153069

[b14] Lyko F. & Maleszka R. Insects as innovative models for functional studies of DNA methylation. Trends Genet 27, 127–131 (2011). 21288591 10.1016/j.tig.2011.01.003

[b15] Lyko F. *et al.* The Honey Bee Epigenomes: Differential Methylation of Brain DNA in Queens and Workers. Plos Biol 8, e1000506 (2010). 21072239 10.1371/journal.pbio.1000506 PMC2970541

[b16] Herb B. R. *et al.* Reversible switching between epigenetic states in honeybee behavioral subcastes. Nature Neuroscience 15, 1371–1373 (2012). 22983211 10.1038/nn.3218 PMC3518384

[b17] Miklos G. L. G. & Maleszka R. Epigenomic communication systems in humans and honey bees: From molecules to behavior. Horm Behav 59, 399–406 (2011). 20594964 10.1016/j.yhbeh.2010.05.016

[b18] Wang Y. & Leung F. C. C. In Silico Prediction of Two Classes of Honeybee Genes with CpG Deficiency or CpG Enrichment and Sorting According to Gene Ontology Classes. J Mol Evol 68, 700–705 (2009). 19466376 10.1007/s00239-009-9244-3

[b19] Tusnady G. E., Simon I., Varadi A. & Aranyi T. BiSearch: primer-design and search tool for PCR on bisulfite-treated genomes. Nucleic Acids Research 33, e9 (2005). 15653630 10.1093/nar/gni012 PMC546182

[b20] Warnecke P. M. *et al.* Identification and resolution of artifacts in bisulfite sequencing. Methods 27, 101–107 (2002). 12095266 10.1016/s1046-2023(02)00060-9

[b21] Lin P., Foret S., Wilson S. R. & Burden C. J. Estimation of the methylation pattern distribution from deep sequencing data. BMC Bioinformatics 16, 145 (2015). 25943746 10.1186/s12859-015-0600-6 PMC4428226

[b22] Ji L. *et al.* Methylated DNA is over-represented in whole-genome bisulfite sequencing data. Front Genet 5, 341 (2014). 25374580 10.3389/fgene.2014.00341 PMC4204604

[b23] Teleman A. A. Privileged Signaling for Brain Growth. Cell 146, 346–347 (2011). 21816269 10.1016/j.cell.2011.07.010

[b24] Patel A. *et al.* The Making of a Queen: TOR Pathway Is a Key Player in Diphenic Caste Development. Plos One 2, e509 (2007). 17551589 10.1371/journal.pone.0000509 PMC1876819

[b25] Grandison R. C., Piper M. D. W. & Partridge L. Amino-acid imbalance explains extension of lifespan by dietary restriction in Drosophila. Nature 462, 1061–1064 (2009). 19956092 10.1038/nature08619 PMC2798000

[b26] Alvarado S., Rajakumar R., Abouheif E. & Szyf M. Epigenetic variation in the Egfr gene generates quantitative variation in a complex trait in ants. Nat Commun 6, 6513 (2015). 25758336 10.1038/ncomms7513

[b27] Maleszka R., Mason P. H. & Barron A. B. Epigenomics and the concept of degeneracy in biological systems. Brief Funct Genomics 13, 191–202 (2014). 24335757 10.1093/bfgp/elt050 PMC4031454

[b28] Wojciechowski M. *et al.* Insights into DNA hydroxymethylation in the honeybee from in-depth analyses of TET dioxygenase. Open Biol 4, 140110 (2014). 25100549 10.1098/rsob.140110 PMC4150289

[b29] Evans J. D. *et al.* Standard methods for molecular research in Apis mellifera. J Apicult Res 52, 1–30 (2013).

