# Supplementary material for: EGFR gene methylation is not involved in Royalactin controlled phenotypic polymorphism in honey bees
Source: Sci Rep. 2015 Sep 11;5:14070. doi: 10.1038/srep14070 (PMC4566103; doi:10.1038/srep14070)
Supplement: Supplementary Information [file srep14070-s1.pdf]

Supplementary Information File

Article title:

EGFR gene methylation is not involved in Royalactin controlled phenotypic polymorphism in honey bees

Authors:

Kucharski, R., Foret, S., Maleszka, R

Table S1

Primer sequences used to generate EGFR bisulfite amplicons used in this study. Cloning adaptors routinely added to the nested PCR primers are underlined.

Primary PCR	EGFR A1 F1	5′-GAGGAAGAATATTGTTTAAGTTTA
	EGFR A1 R1	5′-AAACACCTACCCTACCAACACTAA
Nested PCR	EGFR A1 F2	5′-GCAGAATTCGATGTGTTAGATGTAGAATTTGGA
	EGFR A1 R2	5′-CGCAAGCTTAATACTTCTAACAATTCTCAAACC
Primary PCR	EGFR A2 F1	5′-TAATTGGGAGGAAATAATTATAGG
	EGFR A2 R1	5′-AACCAATTTCATTATTTCAATTAT
Nested PCR	EGFR A2 F2	5′-GCAGAATTCGGTTTGAGAATTGTTAGAAGTATT
	EGFR A2 R2	5′-CGCAAGCTTTATATACATTTCCCAATACATTTC

Figure S1

Corrected EGFR gene model showing the region used for methylation analysis in this study. Blue: annotation of Apis mellifera LG6 (accession NC_007075.3), nucleotides 3,042,121 to 3,043,054. Exon numbering reflects the presence of additional exons identified by analysing RNAseq data. The 5' region of exon 5 and the adjacent intron sequence, aligning with a 117 bp stretch of Ns in the genome assembly 4.5 (LG6, NC_007075.3, nucleotides 3,042,143 to 3,042,259), were reconstructed from RNAseq and homology data following a failure to identify the original forward primer using the coordinates in the Kamakura paper (Supplementary Information, page 18, paragraph "DNA extraction and bisulfite treatment"). The placement of Kamakura's CpG map based on Figure S34 (yellow) is a guesstimate as no coordinates were supplied with that figure. We aligned the beginning of Exon 1 with the ATG corresponding to the first methionine of AmEGFR protein in Figure S21. This protein, as well as the NCBI EGFR RefSeq model (XP_006560089.1), is truncated by circa 170 amino acids compared with the Drosophila EGFR orthologue (FlyBase EGFR-PA) as shown in the figure. The Kamakura first region bisulfite fragment (orange) is aligned using bisulfite primers coordinates after correction. EGFR amplicons A1 and A2 are marked in green.

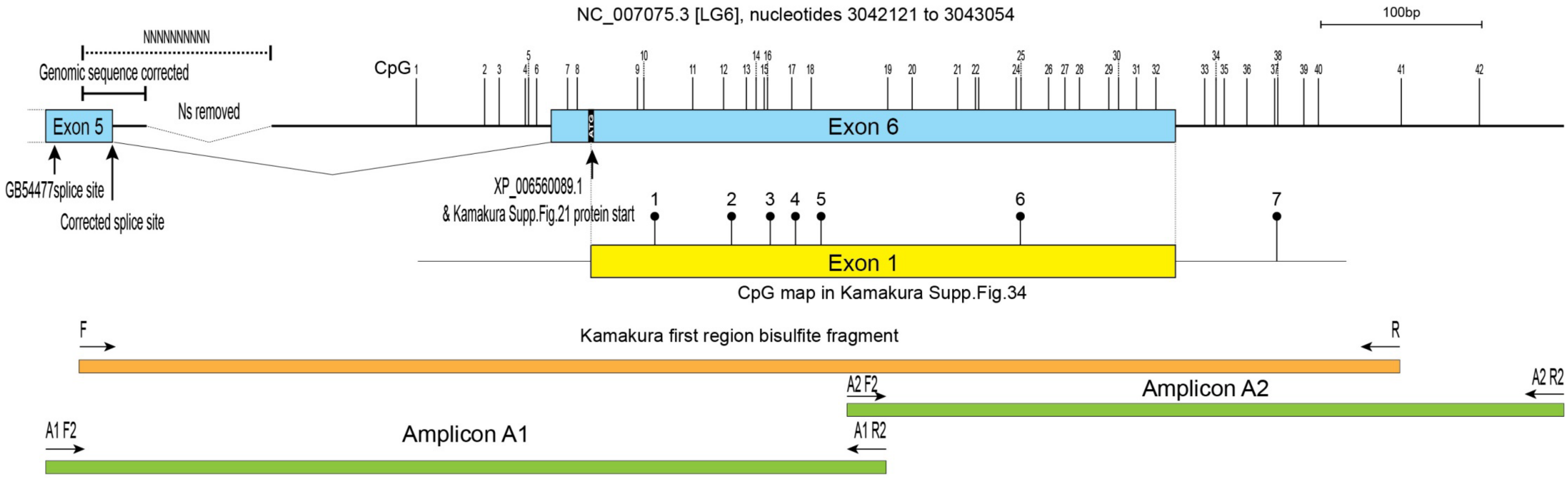
NC_007075.3 [LG6], nucleotides 3042121 to 3043054
100bp
NNNNNNNNNN
Genomic sequence corrected
Ns removed
Exon 5
CpG
Exon 6
ATG
GB54477splice site
Corrected splice site
XP_006560089.1
& Kamakura Supp.Fig.21 protein start
Exon 1
CpG map in Kamakura Supp.Fig.34
F
R
Kamakura first region bisulfite fragment
A2 F2
Amplicon A2
A2 R2
A1 F2
Amplicon A1
A1 R2
